# Assessing the Impact of a Virtual Reality Cognitive Intervention on Tennis Performance in Junior Tennis Players: Pilot Study

**DOI:** 10.2196/66979

**Published:** 2025-02-07

**Authors:** Joaquin A Anguera, Aleem Choudhry, Michael Seaman, Dominick Fedele

**Affiliations:** 1 Neuroscape Department of Neurology and Psychiatry, Weill Institute for Neurosciences University of California, San Francisco San Francisco, CA United States; 2 Mastermind Sports Naperville, IL United States

**Keywords:** executive function, serious games, cognitive training, performance enhancement, athletes, sport, pilot study, VR, virtual reality, serious game, tennis, adolescents, teenagers, youth, randomized controlled trial, players

## Abstract

**Background:**

There is evidence that cognitive training interventions can positively impact executive functions, and that some studies have demonstrated that athletes typically exhibit greater accuracy and faster response times on select cognitive tasks. While the engagement of executive functions is suggested to be part of high-level sporting activities, it is unclear whether such training approaches could directly benefit athletic performance.

**Objective:**

The objective of this study was to evaluate the impact of a combined virtual reality (VR)– and tablet-based cognitive training intervention on adolescent tennis players’ performance. Here, we examined differences in Universal Tennis Rating (UTR) between players who supplemented their regular tennis training with a cognitive training intervention and a group that continued regular tennis training alone. This custom cognitive training program targeted specific cognitive control abilities including attention, working memory, and goal management.

**Methods:**

Data were collected from a cohort of tennis players in a randomized controlled trial design led by the dedicated research team. Participants (N=23, age: mean 14.8, SD 2.4 years) from the Czech Lawn Tenis Klub (Prague, Czech Republic) were invited to participate in this study. These individuals were randomized into an intervention + training-as-usual group (n=13) or training-as-usual group (control group; n=10), with the change in UTR score being the primary metric of interest.

**Results:**

There was no difference in UTR between the 2 groups at baseline (intervention: mean 8.32, SD 2.7; control: mean 7.60, SD 2.3). Following the treatment period, individuals in the intervention group showed a significant improvement in their UTR (an increase of 0.5; t_12_=4.88, *P*<.001) unlike the control group (an increase of 0.02; *t*_9_=1.77, *P*=.12). On comparing the change in UTR (posttraining UTR minus pretraining UTR) attained by each group, we found that the intervention group had a 38% greater improvement in UTR than the control group. An analysis of covariance revealed a significantly greater improvement in UTR for the intervention group than for the control group (*F*_1,20_=8.82, *P*=.008).

**Conclusions:**

The present findings suggest that training cognitive abilities through an immersive visual platform may benefit athletic performance, including tennis. Such a result warrants careful consideration, given the known difficulties in evidencing far transfer not only in cognitive studies but also in athletic activities. These preliminary pilot findings suggest that the Mastermind Cognitive Training program may be a viable tool for supplementing athletic training practices, although this result warrants further investigation and replication. However, many questions remain unanswered, and further work is needed to better understand the potential utility and mechanisms underlying potential effects of such a platform.

## Introduction

The engagement of cognitive control abilities, defined here as attention, working memory, and multitasking [1], is thought to be a critical part of high-level athletic performance. This idea stems from research showing that both amateur and elite athletes typically exhibit greater accuracy and faster response times on select cognitive tasks [2,3], and that one’s underlying cognitive abilities can predict future athletic achievement [4-6]. However, the existing literature on whether enhancing these abilities through cognitive training can improve athletic performance is limited [7-9]. Numerous examples in the cognitive training literature provide reasons why so-called “far transfer” (eg, the ability to apply learning from one context to a different, dissimilar context [10]) is rarely observed [11,12]. However, those positive findings following the use of specific cognitive training platforms suggest that such an approach could possibly be beneficial [13-16]. Such positive effects have been observed in those platforms using closed-loop algorithms, an approach that changes the level of difficulty on a trial-by-trial basis to challenge a given individual appropriately for a given training experience [1,17-19]. This approach has shown generalizable benefits to not only untrained tasks assessing cognitive abilities (including attention, working memory, and multitasking) [16,20-24] but also so-called “real-world” constructs including mood [14,15,25-27] and even academic abilities [28]. Here, we describe the preliminary findings of a proof-of-concept randomized controlled study involving advanced adolescent tennis players who supplemented their regular tennis training with a cognitive training intervention in comparison to a group that continued regular tennis training alone.

## Methods

### Ethical Considerations

For this project, all study procedures were conducted in accordance with protocols approved by the Fakultní Nemocnice Brno (Brno, Czech Republic; 14-110123/EK). Participant assent and parental consent were obtained prior to study participation in person by the study team. All study data were deidentified to ensure privacy and confidentiality protections were in place. No compensation was given for participating in this study.

### Participants

A total of 59 tennis players (age: mean 16.7, SD 3.6 years; 34 male) from the Czech Lawn Tenis Klub (Prague, Czech Republic) were invited to participate in this study (and provided a full overview of the study procedures and time requirements). See Multimedia Appendix 1 for more details.

### Intervention

**Figure 1:**
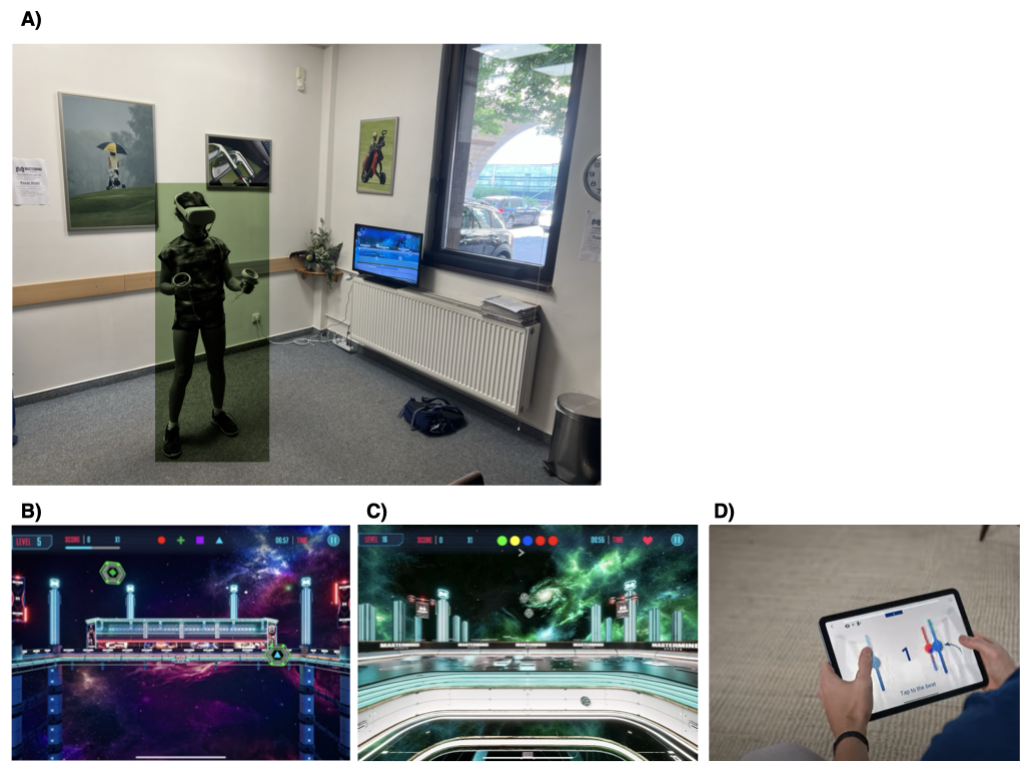
The Mastermind Cognitive Training program. (A) Participant training during the Mastermind Cognitive Training program using virtual reality. Participants were tennis players from the Czech Lawn Tenis Klub (Prague, Czech Republic) who trained between January and November 2023. Participants used VR hand-held controllers to respond to stimuli presented in a video game–like environment, in some cases “shooting” specific target stimuli while avoiding others or trying to catch certain colored targets in a given order stimuli while avoiding nontargets. (B) the virtual reality attention module. (C) The virtual reality multitasking module. (D) The tablet rhythm training module where participants tapped in a rhythmic fashion to stimuli presented on the screen.

The Mastermind Cognitive Training program used a virtual reality (VR) headset (Oculus Quest 2 by Meta) to deliver a custom cognitive training program targeting specific cognitive control abilities including attention, working memory, and goal management. The VR component comprised 3 modules, each focusing on a different aspect of cognitive control: a multiple object–tracking module aimed at engaging attention (with increasing distraction), a spatial span module to engage working memory, and a visual search module with task switching requirements to engage multitasking abilities. In each case, participants used VR hand-held controllers (Figure 1A) to respond to stimuli presented in a video game–like environment, in some cases “shooting” specific target stimuli while avoiding others (Figure 1B), or trying to catch certain colored targets in a given order stimuli while avoiding nontargets (Figure 1C). Each module was adaptive, adjusting task difficulty based on performance. In addition, participants completed 20 minutes of tablet-based training (iPad) on tasks related to rhythmic timing (where participants were asked to tap on the screen to the beat of music being played [28-31]; Figure 1D). The intervention group was asked to complete 24 program sessions over the course of 10 weeks, with each session consisting of 15 minutes of VR exercises and 20 minutes of tablet-based exercises, totaling approximately 14 hours of training. See Multimedia Appendix 1 for more details.

### Measure

The primary outcome measure was the change in Universal Tennis Rating (UTR), a scale from 1.00 (eg, a beginner player) to 16.50 (eg, a professional player; for context, the current #1 player in the world is rated 16.05), which promotes fair and competitive play [32]. Each player’s UTR was collected before training and after the completion of the 24-session training period, which an interassessment interval of approximately 4 months. See Multimedia Appendix 1 for more details about this measure.

## Results

**Figure 2:**
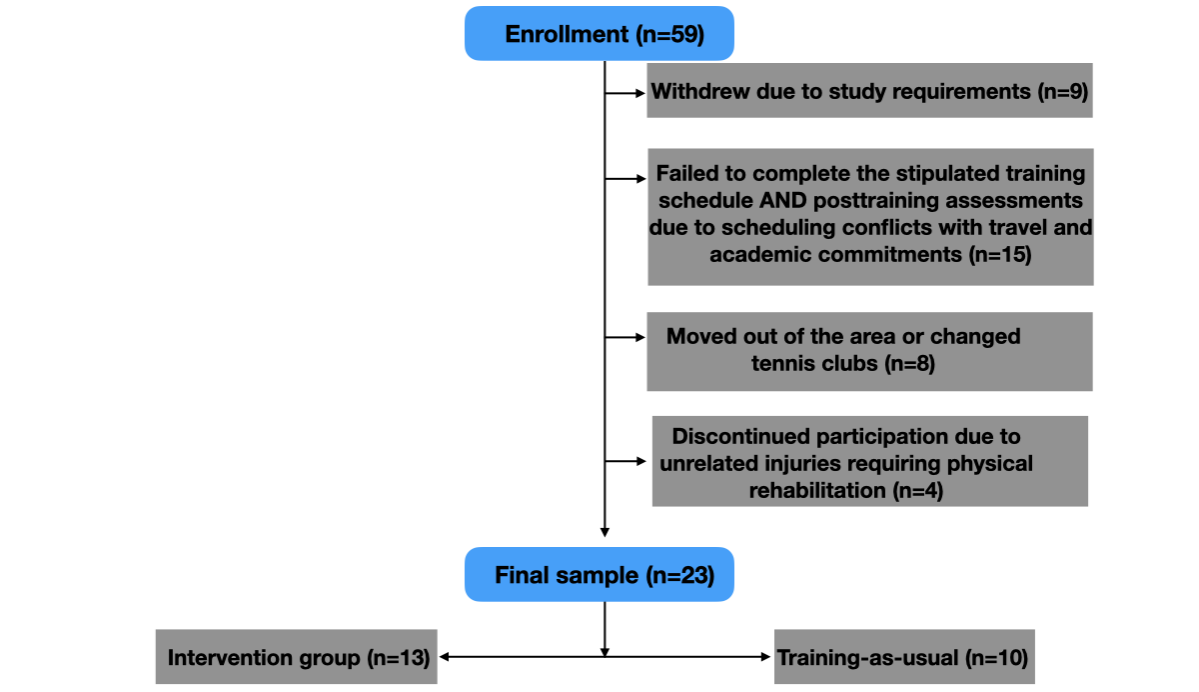
CONSORT (Consolidated Standards of Reporting Trials) flow diagram.

**Figure 3:**
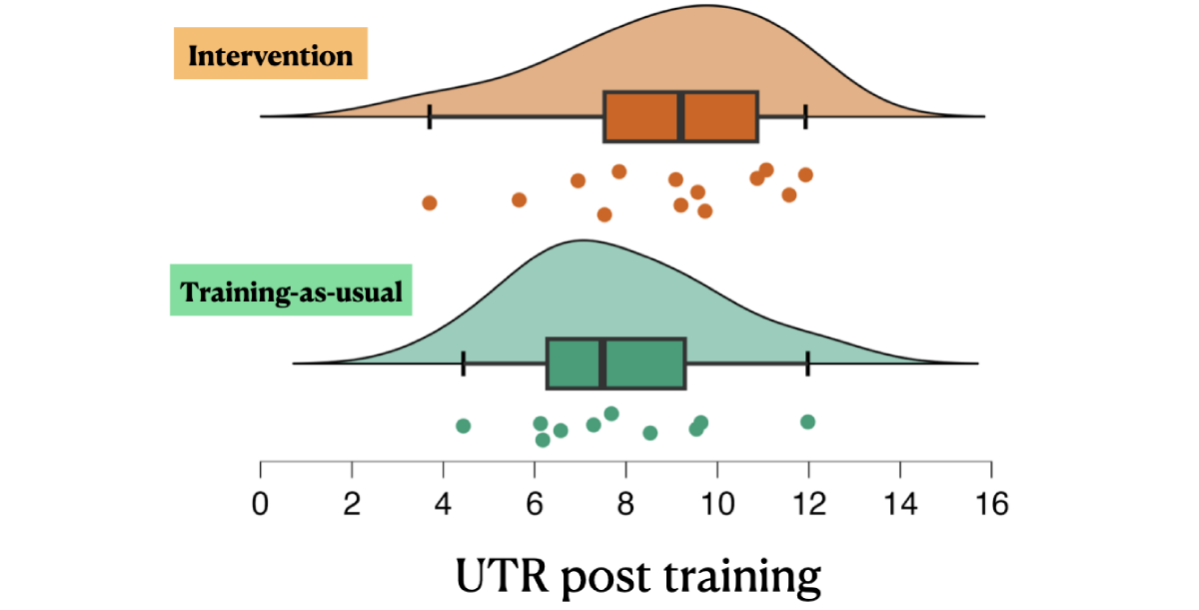
Universal Tennis Rating (UTR) by group. The UTR is a scale from 1.00 (eg, a beginning player) to 16.50 (eg, a professional player), which promotes fair and competitive play. Each player’s UTR was recorded before training and after the completion of the 24-session training period (approximately 4 months). Posttraining UTR scores for the intervention and training-as-usual groups visualized via a raincloud plot with difference-adjusted 95% CIs of the mean. An analysis of covariance (controlling for pretraining performance) revealed that the improvement observed by the intervention group was significantly greater than that of the training-as-usual group (*F*_1,20_=8.82, *P*=.008).

Of the 59 consenting individuals, 9 withdrew upon learning of the study requirements, 15 failed to complete the stipulated training schedule and posttraining assessments due to scheduling conflicts with travel and academic commitments, 8 opted out due to moving out of the area or changing tennis clubs, and 4 discontinued participation due to unrelated injuries requiring physical rehabilitation. Thus, the final sample that completed the stipulated training and outcome assessments consisted of 23 participants: 13 in the intervention group (3 female, aged mean 15.0, SD 2.4) and 10 (7 female, aged mean 14.6, SD 2.5) in the training-as-usual group (see Figure 2 for the CONSORT [Consolidated Standards of Reporting Trials] diagram and Multimedia Appendix 2 for CONSORT checklist). To test for training effects on UTR (Table 1), we conducted an analysis of covariance (ANCOVA) with posttraining UTR as the dependent variable, pretraining UTR as the covariate, and the group as the fixed factor as in our previous work [14,20,33,34]. The analysis revealed a significant group difference in UTR improvement over time (*F*_1,20_=8.82, *P*=.008; Figure 3), indicating that the UTR of the intervention group improved significantly more than that of the control group. On comparing the change in UTR (posttraining UTR minus pretraining UTR) attained by each group, the intervention group had a 38% greater improvement in UTR than the control group. Post hoc analysis of the within-group changes in UTR using 2-tailed, paired-samples *t* tests for each group separately to test for significant differences between each testing session revealed a significant improvement by the intervention group (increase of 0.50; t_12_=4.88, *P*<.001) unlike the control group (increase of 0.20; *t*_9_=1.77, *P*=.12). Critically, there was no difference in UTR at baseline between the 2 groups (independent-samples *t* test: t_21_=.68, *P*=.50).

**Table 1:** Universal Tennis Rating (UTR) by group and timepoint.

	Pretraining UTR, mean (SD)	Posttraining UTR, mean (SD)	Change (posttraining UTR–pretraining UTR)
Intervention	8.32 (2.7)	8.83 (2.4)	0.50 (0.37)
Treatment-as-usual	7.60 (2.3)	7.79 (2.2)	0.20 (0.37)

## Discussion

### Principal Findings

The primary goal of this proof-of-concept pilot study was to investigate the effects of the Mastermind Cognitive Training program in adolescent tennis players. These findings suggest that supplementing regular training with cognitive training can lead to meaningful improvements in real-world athletic performance. It has been suggested that visual-based training (including the use of VR and/or tablet platforms) is more likely to benefit trained athletes than novices [35]. Indeed, this theory has empirical support: “real-world” improvements have been observed, including performance in college-level baseball players [36,37] as well as table tennis performance [38] when coupling such training with their regular sports training [38]. In conjunction with those studies, the present findings suggest that training cognitive abilities through an immersive visual platform may benefit performance on athletic endeavors, including tennis. Such a result warrants careful consideration, given the known difficulties in evidencing far transfer not just in cognitive studies, but especially in athletic activities [7]. That said, our findings do align with those of the few positive studies demonstrating that cognitive training can result in some form of on-field improvements [8,9]. See Multimedia Appendix 1 for a further discussion of practical considerations.

### Limitations

Several limitations should be considered with the present findings. First, the most important factor to consider with this study is the small sample size, especially considering the large number of individuals who did not complete the study. It should be noted that this study was not designed to be anything more than a pilot study intended to gauge the feasibility of implementing the Mastermind Cognitive Training program following the unexpected opportunity to work with adolescent tennis players. Future work must involve a larger trial of participants to replicate these findings to provide supporting evidence for the present interpretations. Second, the very real possibility exists that participation in the intervention generated a placebo effect through the novel use of a VR platform. Given the pervasive problem of placebo effects in such work [39], it is critical that future endeavors account for these effects in a carefully designed manner. Finally, it is unknown what aspect of the training program led to the improvement in UTR. The absence of cognitive control outcome measures to assess whether, and to what extent, this training program led to cognitive changes hinders a deeper understanding of what possibly drove the differential improvement in UTR. Future work is warranted to better understand the potential underlying mechanisms driving such change.

### Conclusions

This exploratory proof-of-concept study investigated the potential benefits of a novel cognitive intervention platform in a sample of healthy adolescent tennis athletes. It represents the first step toward a number of data-driven studies demonstrating the utility and benefits of the use of this platform. These pilot findings suggest that this platform may be beneficial for adolescent tennis athletes looking to improve their sporting performance by supplementing their existing training protocols with a targeted cognitive training experience. However, many questions remain unanswered, and further work is needed to better understand the potential utility and mechanisms underlying potential effects of such a platform.

## Appendix

Multimedia Appendix 1 Additional material.

Multimedia Appendix 2 CONSORT-EHEALTH (V 1.6.1) checklist.

## Abbreviations

ANCOVA: analysis of covariance
CONSORT: Consolidated Standards of Reporting Trials
UTR: Universal Tennis Rating
VR: virtual reality

## Data Availability

﻿The datasets generated during and/or analyzed during this study are available from the corresponding author upon reasonable request.
