# Supplementary material for: Assessing the Impact of a Virtual Reality Cognitive Intervention on Tennis Performance in Junior Tennis Players: Pilot Study
Source: JMIR Form Res. 2025 Feb 7;9:e66979. doi: 10.2196/66979 (PMC11845879; doi:10.2196/66979)
Supplement: Multimedia Appendix 1 [file formative_v9i1e66979_app1.docx]

**SUPPLEMENTAL MATERIALS**

**Additional background on participant participation**

Located on Štvanice Island, this is the oldest and most prestigious tennis club in the Czech Republic, with membership in the club offered to the general public for both competitive and amateur tennis. Recruitment for this study entailed a combination of posted flyers and word of mouth from our study team (who have membership at this club). All outcome data and training measures took place at the tennis club as led by the study team, who provided all necessary equipment for study participation, with the study beginning in January of 2023 and data collection completed in November of 2023. ﻿This study was not registered before patient recruitment as this study was implemented to gauge the feasibility and potential efficacy of using this type of approach in these populations as a gauge for a subsequent large-scale intervention trial. Consented individuals were equally allocated by age rages and genders into 6 subgroups, and within these subgroups participants were randomized using a pre-generated randomization sequence to each potential group (intervention + training-as-usual group or a training-as-usual group).

**Training protocols and additional details about the intervention**

Each session was completed at the Czech Lawn Tenis Klub, and was supervised by one of the study team members to ensure quality data collection as well as ensure the safety of the training participants. A typical training day involved tennis practice twice a day, and then in between their tennis training those individuals in the intervention group would come to a dedicated room to complete their Mastermind Training. The room could house up to a maximum of 4 participants on VR and 4 on tablet, but typically there were 1-2 participants at a time.  While most of the sessions took place on-site, occasionally if a player was traveling extensively for a tournament, they were allowed to take a VR headset and iPad with them to complete their training sessions outside of the club after learning how to navigate the technology and applications. This aligned with the overarching goal of making this program scalable and accessible, given that the program was designed to be self-explanatory.

The Mastermind Cognitive Training program™ utilized a Virtual Reality (VR) headset (Oculus Quest 2 by Meta™) to deliver a custom cognitive training program targeting specific cognitive control abilities including attention, working memory, and goal management. The decision to deploy the cognitive training in virtual reality was based on previous work demonstrating that this particular environment ﻿allows users to perceive experiences in a deeper manner than 2D (that is, have greater engagement)[1-3], with subsequent work demonstrating that task performance in a VR experience is enhanced as compared to the performance of the same task in a 2D space like a computer screen[4]. The present intervention was created to enhance both cognitive control abilities as well as visual processing abilities, as these facets are common to a number of different sports. To be clear, this platform was not designed specifically for tennis, but to positively impact individuals across numerous sporting domains (e.g. baseball, football, soccer, etc.) where rapid decision making is required with respect to processing visual information. Additional details and images of the training program can be found at mastermindsports.com, with videos of the VR tasks shown at (<https://youtu.be/pJ0IPutvPMU)>.]

**UTR Measure**

#### The primary outcome measure was the change in Universal Tennis Rating (UTR), a scale from 1.00 (e.g. a beginning player)-16.50 (e.g. a professional player; for context, the current #1 player in the world is rated 16.05) that promotes fair and competitive play[5]. Each player’s UTR was collected prior training and after the completion of the 24-session training period, which an inter-assessment interval of approximately 4 months. A player’s UTR is the weighted average of up to 30 of their most recent match ratings within the past 12-month period. The UTR calculation involves: 1) the difference in UTR between opponents, and 2) match competitiveness, as determined by the percentage of games won by each player. A given match is also assigned a weight, as determined by 1) match format length (longer has greater weight), 2) closeness of UTR ratings of the opponent, 3) UTR reliability (greater reliability has greater weight), and 4) a recency bias towards recent matches.

**Practical considerations**

It is worth noting the practical aspects impacting the logistics of this training. 9 of the 59 participants felt at the outset that the time requirements for the study (in addition to their regular training schedule) were too arduous given their existing schedule, with 15 others withdrawing for related reasons. This level of attrition warrants further scrutiny in future work to determine whether that the time requirements designed here (30-minute sessions totaling 14 hours of training over 10 weeks) could potentially be adjusted to promote greater participation. Considerations are also warranted regarding the feasibility of delivering the training given the present use of a trained facilitator for most training sessions. While having a research proctor was warranted here for this formative work to ensure the safety of the participants and quality of data collected, the ability to self-assess and self-administer such a platform would lend itself to any such platform being truly scalable.

References

1. Slobounov, S.M., et al., *Modulation of cortical activity in 2D versus 3D virtual reality environments: An EEG study. .* International Journal of Psychophysiology, 2015. **95**: p. 254–260.

.

2. Kober, S.E., J. Kurzmann, and C. Neuper, *Cortical correlate of spatial presence in 2D and 3D interactive virtual reality: An EEG study. .* International Journal of Psychophysiology, 2012. **83**: p. 365–374.

3. Yehene, E., N. Meiran, and N. Soroker, *Task alternation cost without task alternation: measuring intentionality.* Neuropsychologia, 2005. **43**(13): p. 1858-69.

4. Li, G., et al., *Enhanced Attention Using Head-mounted Virtual Reality.* J Cogn Neurosci, 2020. **32**(8): p. 1438-1454.

5. Sports, U. *How UTR Rating Works*. 2023 9/25/2024]; Available from: <https://www.utrsports.net/blogs/news/how-utr-works>.
